# Dietary and Physical Activity Behaviors in Women with Polycystic Ovary Syndrome per the New International Evidence-Based Guideline

**DOI:** 10.3390/nu11112711

**Published:** 2019-11-08

**Authors:** Annie W. Lin, Maryam Kazemi, Brittany Y. Jarrett, Heidi Vanden Brink, Kathleen M. Hoeger, Steven D. Spandorfer, Marla E. Lujan

**Affiliations:** 1 Department of Preventative Medicine, Feinberg School of Medicine, Northwestern University, Evanston, IL 60611, USA; 2 Division of Nutritional Sciences, Cornell University, Ithaca, NY 14853, USA; 3 Department of Obstetrics and Gynecology, University of Rochester Medical Center, Rochester, NY 14623, USA; 4 Ronald O. Perelman and Claudia Cohen Center for Reproductive Medicine, Weill Cornell Medicine, New York, NY 10065, USA

**Keywords:** diet, exercise, polycystic ovary syndrome, healthy lifestyle, nutritional assessment

## Abstract

Lifestyle modifications are recommended as first-line therapy in polycystic ovary syndrome (PCOS). However, usual dietary and physical activity (PA) behaviors of women with PCOS remain uncertain, likely owing to controversy in diagnostic criteria. Our objective was to contrast the usual dietary and PA behaviors of women with PCOS (*n* = 80) diagnosed by the 2018 International Evidence-based Guideline for the Assessment and Management of PCOS to that of controls (*n* = 44). Study outcomes were dietary intake, diet quality (Healthy Eating Index-2015), and PA (questionnaire, waist-worn accelerometers). Women with PCOS met the acceptable macronutrient distribution ranges for carbohydrate, fat, and protein, but did not meet the recommended dietary reference intakes for vitamin D (mean (95% confidence interval); 6 (5–7) μg/d), vitamin B9 (275 (252–298) μg/d), total fiber (24 (22–26) g/d), or sodium (4.0 (3.6–4.4) g/d). Women with PCOS also met the US recommendations for PA. No differences were detected in dietary intake, diet quality, or PA levels between groups (*p* ≥ 0.11). In conclusion, women with and without PCOS have comparable dietary and PA behaviors. A lack of unique targets for dietary or PA interventions supports the position of the new guideline to foster healthy lifestyle recommendations for the management of PCOS.

## 1. Introduction

The recent International Evidence-based Guideline for the Assessment and Management of Polycystic Ovary Syndrome (PCOS) has emphasized the importance of diet and physical activity for managing the signs and symptoms of PCOS and preventing the metabolic complications associated with the syndrome [1]. The recommendations support weight management across the life course for all women with PCOS, including weight loss for women with comorbid overweight or obesity and the prevention of weight gain for women within a healthy weight range. A focus on weight management practices is predicated based on substantial evidence that obesity worsens reproductive and metabolic profiles in PCOS [2].

Up to 80% of women with PCOS present with overweight or obesity [3,4,5]. Although preliminary evidence suggests that women with PCOS are more susceptible to weight gain [6], controversy exists on whether dietary and physical activity behaviors contribute to the development of PCOS [4,6]. Poor diet has been associated with individual PCOS features, such as hyperandrogenemia and polycystic ovaries [7,8,9,10,11], as well as self-reported infertility [12,13,14,15,16]. However, whether women with PCOS consume poorer diets and/or participate in shorter intervals of physical activity, which could contribute to a propensity for weight gain, in this clinical population remain unclear [17]. The possibility of excessive energy intake in women with PCOS is controversial. Some studies have reported similar energy intake and physical activity between women with and without PCOS [15,18,19,20], while others have identified a positive energy balance in women with PCOS, secondary to excessive caloric intake and sedentary lifestyle behaviors [10,13,21,22]. Improved diet quality has also been observed in women with PCOS, albeit in conjunction with increased energy intake and longer sitting intervals [14]. However, all [12,13,15,19] but one study [14] have identified no differences in sedentary behaviors or physical activity between women with and without PCOS. Collectively, little can be concluded regarding the existence of unique diet and physical activity targets for intervention in women with PCOS.

The mixed evidence regarding usual dietary and physical activity behaviors in women with PCOS may stem, in part, from variability across studies in the criteria used to identify the syndrome [9]. Studies of lifestyle behaviors in the United States [15,18] have used the National Institutes of Health (NIH) criteria, which do not consider polycystic ovarian morphology as a diagnostic feature. Such an approach narrows the phenotypic spectrum of PCOS and, therefore, limits the comparability and generalizability of any findings [23]. A dependence on self-reported histories of PCOS and differences in demographics across studies has added further variability to the evidence. Recently, the International Evidence-based Guideline for the Assessment and Management of PCOS recommended the assessment of ovarian morphology, hyperandrogenism, and oligo-amenorrhea for the clinical diagnosis of PCOS. In addition to variability in diagnostic definitions, technical challenges related to the reliable assessment of androgen status [24], ovarian morphology [25], and lifestyle behaviors [9] have also contributed to the conflicting data. In the case of the lifestyle measures, previous studies have used a variety of instruments to collect dietary (e.g., food records, recalls, questionnaires) and physical activity information (e.g., questionnaires, interviews). Notably, physical activity data have mainly been based on self-report, which can be biased by the recall period or social desirability [26]. Objective tools to measure physical activity (e.g., accelerometer and pedometer) have rarely been implemented in PCOS research [27,28,29] but could help to clarify the magnitude of sedentary lifestyle behaviors in women with PCOS [9].

To our knowledge, no effort has been made to evaluate the lifestyle behaviors of women with PCOS using the updated diagnostic criteria supported by the 2018 evidence-based guideline. This evaluation is particularly important as the recent guideline concluded there is no or limited evidence to support a specific dietary or physical activity regimen to improve health outcomes in women with PCOS [1]. Tailored lifestyle modifications that account for existing dietary intake and physical activity levels may be critical for the successful adoption and sustainability of lifestyle changes in this clinical population. To address this knowledge gap, we investigated the usual dietary intake, diet quality, and physical activity levels of a well-defined cohort of women with PCOS in the United States using the latest criteria to define PCOS, and compared their lifestyle behaviors against controls without PCOS. Differences in lifestyle behaviors of women with PCOS compared to healthy women could serve as unique targets of intervention for this clinical population.

## 2. Materials and Methods

### 2.1. Study Design and Setting

The present case-control study represents a cross-sectional analysis of women recruited from New York (NY) state to one of six study protocols that prospectively evaluated the lifestyle behaviors in women of reproductive age. Women were recruited between January 2013 and July 2018 using paper and electronic advertisement circulated in our local campuses, clinics, and public community spaces: (1) Human Metabolic Research Unit, Division of Nutritional Sciences, Cornell University, Ithaca, NY; (2) Strong Fertility Center, Department of Obstetrics and Gynecology, University of Rochester Medical Center, Rochester, NY; or (3) Center for Reproductive Medicine, Weill Cornell Medicine, New York, NY.

The Institutional Review Boards at Cornell University, University of Rochester, and Weill Cornell Medicine approved the research protocols (ClinicalTrials.gov: NCT01927432, NCT01927471, NCT01785719, NCT01859663, and NCT1410015577). All procedures were conducted in compliance with the World Medical Association Declaration of Helsinki, and the Guidelines of the International Conference on Harmonization on Good Clinical Practice. All participants provided written, informed consent at study enrollment.

Women were eligible to participate if they were aged 18 to 45 years old and exhibited no symptoms of the menopausal transition (i.e., no recent changes in menstrual patterns or abnormal elevations in follicle stimulating hormone). Exclusion criteria were the use of appetite- or weight-affecting or insulin-sensitizing medications within two months of study participation, and the presence of medical conditions known to interfere with reproductive or metabolic function (besides PCOS), including diabetes mellitus, hyperprolactinemia, untreated thyroid dysfunction, and premature ovarian failure. None of the included participants were actively seeking or were involved in fertility therapy. Of the 127 women deemed eligible for the present study, three women were ultimately excluded due to implausible energy intakes, resulting in a final study population of 124 women.

### 2.2. Definition of PCOS

Participants were evaluated either during the early follicular phase of the menstrual cycle (in women reporting regular menstrual cycles or the use of hormonal contraception) or at a random time when no dominant follicles or corpora lutea were present (in women reporting irregular menstrual cycles). PCOS was diagnosed according to the recommended thresholds of the 2018 International Evidence-based Guidelines for the Assessment and Management of PCOS [1] and complied with the 2003 Rotterdam consensus criteria [30] of the presence of two or more features of: (1) Oligo-amenorrhea, (2) hyperandrogenism, and, (3) polycystic ovarian morphology. Oligo-amenorrhea was defined by a self-reported average menstrual cycle length ≥36 days in the year prior to study enrollment. Evidence of hyperandrogenemia was corroborated with an elevation in at least (1) fasting serum total testosterone concentration (≥2.1 nmol/L); (2) calculated free testosterone (≥0.03 nmol/L); (3) calculated bioavailable testosterone (≥0.7 nmol/L); and/or (4) free androgen (free androgen index (FAI) ≥ 6); thresholds reflected the 95th percentiles of androgen concentrations in an internal reference cohort. Polycystic ovaries were defined by a mean follicle number per ovary (FNPO) ≥ 20 or mean ovarian volume (OV) ≥ 10 mL. A mean follicle number per single cross-section (FNPS) ≥ 9 was used to identify polycystic ovaries if poor image quality prevented the reliable evaluation of FNPO or OV [31]. The control group was comprised of women with menstrual regularity, without a clinical diagnosis of PCOS.

### 2.3. Study Procedures

#### 2.3.1. Clinical Assessment

A standardized reproductive health history and physical examination was completed for all women to assess demographics, anthropometry, and PCOS status. Participants wore light clothing and removed their shoes before anthropometric assessments. Height was measured using a standard stadiometer to the nearest 0.5 cm, and weight was taken using a calibrated digital scale to the nearest 0.1 kg. Body mass index (BMI) was calculated as weight in kilograms divided by height in meters squared. Waist circumference and hips circumference were measured and used to calculate the waist to hip ratio following the World Health Organization Waist Circumference Expert Consultation on waist circumference protocol [32].

#### 2.3.2. Ultrasonographic Assessment

Whole ovaries were scanned from their inner to outer margins in the longitudinal plane using GE Voluson ultrasound machines (E8, S6, S8, or S10 Series; GE Healthcare, Milwaukee, US) with 5–9 MHz or 6–12 MHz multi-frequency transducers. Ultrasound examinations were completed using a standardized protocol across research sites. Two-dimensional cineloops were archived for the offline analysis of mean FNPO and OV using customized imaging software (Sante DICOM Editor, Santesoft LTD, Athens, Greece). Reliable estimates of FNPO (2–9 mm) and FNPS (2–9 mm) were obtained throughout each ovary using the grid system, as previously described [25]. OV was estimated in the largest cross-section of each ovary using the simplified formula for a prolate ellipsoid: 0.5 × (transverse diameter) × (anteroposterior diameter) × (longitudinal diameter) [33].

#### 2.3.3. Biochemical Assessment

Fasting serum concentrations of total testosterone (Brigham Research Assay Core, Boston, MA, US) were measured by liquid chromatography tandem mass spectrometry at a clinical chemistry lab participating in the Centers for Disease Control and Prevention Hormone Standardization Program, as previously described [34]. Serum concentrations of sex hormone binding globulin were measured using chemiluminescence immunoassay (Siemens Medical Solutions Diagnostics, Deerfield, IL, US). FAI [35], free testosterone, and bioavailable testosterone were calculated using validated formulae [36]. Samples were processed for serum and stored at −80 °C until the time of analyses. All inter- and intra-assay coefficients of variation were ≤6.2%, consistent with good assay performance.

#### 2.3.4. Dietary Assessment

Food consumption data was collected using VioScreen™ (Version 2.17; VioCare, Inc., Princeton, NJ, US). VioScreen is an adult-validated, self-administered, web-based food frequency questionnaire (FFQ) that was developed with grant funding from the NIH [36]. Vioscreen FFQ has been used in both research and clinical settings to assess the habitual diet over the past three months and uses graphics with approximately 1200 food images and branching questions that reduce missing foods and respondent burden [36,37]. Nutritional supplement use was also inquired. Further details about the Vioscreen FFQ have been published [38]. Specific nutrient intakes and food data were calculated by processing the FFQ data using the Nutrition Data System for Research software (Version 42; Nutrition Coordinating Center, University of Minnesota, Minneapolis, MN, US) [39].

Diet quality was assessed with the Healthy Eating Index 2015 (HEI-2015). Calculation of HEI-2015 was based on the Department of Agriculture (Washington, US) and the National Cancer Institute, and aligned with the 2015–2020 Dietary Guidelines for Americans [39] and complied with the population ratio method [40]. Briefly, the HEI-2015 consists of 13 food items. The first six items include (1) total vegetables, (2) total fruits, (3) whole fruits, (4) greens and beans, (5) seafood and plant proteins, and (6) total proteins, which can be scored from 0 to 5 points each. The remaining seven items include (7) whole grains, (8) low-fat dairy, (9) fatty acids ratio (polyunsaturated fatty acids plus monounsaturated fatty acids to saturated fatty acid), (10) refined grains, (11) sodium, (12) added sugars, and (13) saturated fats, which can be scored from 0 to 10 points each. For each HEI item, dietary constituents were summed together. For example, the “greens and beans” item was created from the sum of dark green vegetables and legumes (beans and peas). The means of each of the dietary constituents across individuals were computed thereafter, and the appropriate ratios were constructed for the population. Specifically, most food components (except for the fatty acids ratio, added sugars, and saturated fats) were scored on a density basis per 1000 kcal or as a percentage of energy. Four components (sodium, refined grains, added sugars, and saturated fats) were reverse scored (i.e., higher intakes received lower scores). The ratios of the dietary components to 1000 kcal of energy were scored according to the algorithm. Total HEI-2015 scores were computed by aggregating scores across individual dietary components such that total scores ranged from zero (poor diet quality) to 100 (optimal diet quality) [41,42,43].

#### 2.3.5. Physical Activity Assessment

Objective measures of physical activity were obtained with the Actigraph triaxial accelerometer GT3X (27 g; 3.8 cm × 3.7 cm × 1.8 cm) and wGT3X+ (19 g; 4.6 cm × 3.3 cm × 1.5 cm), with a maximum acceleration sampling rate of 50 Hz and without a low frequency extension (Actigraph LLC, Pensacola, FL, US). Participants were asked to wear the accelerometer at the left hip for seven days [44]. Data were included for analysis if the participant wore the accelerometer for at least four days, where an entire day was defined as wear for at least 10 h. Raw data from accelerometers were processed to generate wear minutes from vector magnitude counts using the Sasaki algorithm [45] with an internally developed Excel model. Minutes spent within sedentary, light, moderate, and vigorous activities were reported [46].

Subjective measures of physical activity were obtained with the Women’s Health Initiative Study Physical Activity Questionnaire [47]. Energy expenditure was estimated in metabolic equivalent task (MET) units. One MET is defined as the energy it takes to sit quietly, which is equivalent to approximately 1 kcal/kg/h for an average adult [48]. An estimated MET level was assigned to each type of activity (e.g., walking, mild, moderate, vigorous), as previously described [47,48]. Summary variables (MET-hours/week) were then created by combining frequency, duration, and MET-estimated intensity for that activity.

### 2.4. Statistical Analyses

**Figure 1:**
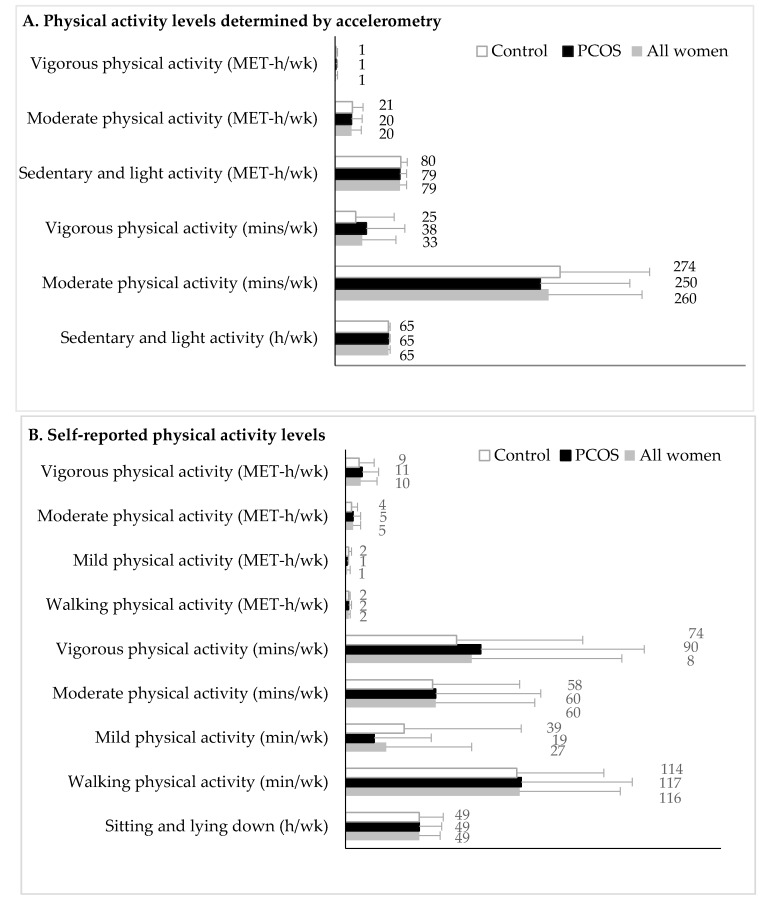
Physical activity levels of women with polycystic ovary syndrome and controls. Physical activity levels measured by accelerometry are shown in Panel **A** (*n* = 82 women; *n* = 48 in the PCOS and *n* = 34 in the control group). Physical activity levels measured by the Women’s Health Initiative Study Physical Activity Questionnaire are shown in Panel **B** (*n* = 101 women; *n* = 62 in the PCOS and *n* = 39 in the control group). Data are expressed as the mean and standard deviation. Physical activity levels were not different between groups in the crude models and after adjusting for age and body mass index differences and the number of comparisons (all: *p* ≥ 0.14). Abbreviations: PCOS, polycystic ovary syndrome; MET, metabolic equivalent task.

Statistical analyses were performed using SPSS version 25.0 (IBM, Armonk, NY, US) and R version 3.6.1. (R Foundation for Statistical Computing, Vienna, Austria). Results are presented as mean (95% confidence interval) or frequency (percentage) for each group, except in Figure 1, where the mean (standard deviation) is reported for clarity. Student’s t-test, Mann–Whitney U, or Chi-square analyses were used to compare demographic and diagnostic features of women with and without PCOS. Dietary intake and physical activity were compared between women with PCOS and controls. Adjusted comparisons were performed on dietary intake, diet quality, and physical activity levels using the analysis of covariance to account for demographic and anthropometric factors, including age and BMI differences, between the groups. Sensitivity analyses were performed to evaluate whether effect estimates changed after excluding women who used metformin and OCP. Multiple testing was corrected by the false discovery rate (FDR) procedure to control the expected proportion of false positives. The standard R function p.adjust was used to adjust *p*-values for multiple testing using the Benjamini–Hochberg method [49], and if there were significant differences between the groups, adjusted *p*-values were reported. Results were considered significant at *p* < 0.05.

## 3. Results

### 3.1. Demographic, Clinical, and Biochemical Characteristics of Women

Participant characteristics are presented in Table 1. Eighty (65%) of the 124 women included in the study had PCOS according to the International Evidence-based Guideline for the Assessment and Management of PCOS. Forty-four women (35%) were included in the control group. Most women (77/124, 62%) were white and within the overweight or obesity BMI ranges (82/124, 66%). Eighty-eight (71%) of all women included in the present study provided information about their education levels. Sixty-one (69%) of these women had a university, college, or associate degree and 27 (31%) had a high school or general education diploma. There were no differences between the education levels of the PCOS and control groups (*p* = 0.11; data not shown). A small proportion of women used metformin (1/124, 0.8%) or oral contraceptive pills (OCPs) (7/124, 6%). Sensitivity analysis confirmed that the inclusion of women taking metformin or OCPs did not alter the results related to dietary intake or physical activity (data not shown). Overall, 49/124 (39.5%) of all women used nutritional supplements, without differences between women in each group (PCOS, 36.3% vs. control, 45.5%; *p* = 0.34). Similarly, there were no differences in the type or dose of consumed nutritional supplements between women with and without PCOS (*p* ≥ 0.27; data not shown).

### 3.2. Dietary Behaviors of Women

Dietary intake and diet quality of women with PCOS and controls are presented in Table 2. Overall, there were no differences in total energy intake between women with and without PCOS. Both groups showed a mean acceptable macronutrient distribution range (AMDR) for carbohydrates (PCOS, 48% vs. control, 49%; *p* = 0.73), fat (PCOS, 36% vs. control, 35%; *p* = 0.79), and protein (PCOS, 16% vs. control, 16%; *p* = 0.57). No differences in dietary intake and quality were observed between groups, after accounting for age and BMI and adjusting *p*-values for the number of comparisons (all: *p* ≥ 0.46; data not shown).

### 3.3. Physical Activity Behaviors of Women

Objective and subjective physical activity are presented in Figure 1. Of the 82 women that provided sufficient data to quantify objective measures of physical activity, 48 had PCOS and 34 were in the control group (Figure 1A). Self-reported measures of physical activity were available for 101 women, 62 with PCOS and 39 in the control group (Figure 1B). No differences were observed in the duration, type, or intensity of physical activity between women with and without PCOS, regardless of whether they had completed both accelerometry and/or the physical activity questionnaire (Supplementary Table S1). No differences in physical activity were observed between groups, after accounting for age and BMI and adjusting *p*-values for the number of comparisons (all: *p* ≥ 0.14; data not shown).

## 4. Discussion

We compared dietary and physical activity behaviors in women with and without PCOS using a well-defined cohort per the most updated diagnostic criteria available. Our data are consistent with the conclusion that women with PCOS consume similar diets and engage in comparable levels of physical activity compared to women without PCOS, despite having a higher BMI. Although our observations did not reveal unique targets for dietary or physical activity interventions in women with PCOS, both groups exhibited inadequate intake of vitamin D, total fiber, vitamin B9, and excessive consumption of sodium, when compared to the US Dietary Reference Intakes. Our observations support the position of the recent evidence-based guideline to promote the adoption and/or maintenance of healthy lifestyle habits of women with PCOS using national recommendations for healthy lifestyle practices.

A lack of differences in dietary intake is consistent with previous reports of comparable energy and macronutrient intakes between women with and without PCOS [15,19]. Our findings also corroborate existing evidence that women with PCOS meet recommended macronutrient distribution ranges [13,14,15,18,19,22]. We noted that our participants exhibited marginally low intakes of dietary fiber (24 g/d), and therefore mirrored the previous observations made by our group and Cutler et al. (2019) in Canadian women [20,50]. Women with PCOS also had excessive sodium intake. While sodium intake was similar to women without PCOS, it exceeded the chronic disease risk reduction intake (CDRR) levels (≤2.3 g/d) and builds on existing evidence of high sodium intake among American [18] and Canadian [50] women with PCOS. Together, our observations highlight the importance of evaluating the fiber and sodium intake of patients with PCOS, particularly in light of their higher propensity for insulin resistance and adverse cardiovascular disease risk profile compared to women without PCOS [51,52].

Women with and without PCOS in the present study also did not meet the US Dietary Recommendations for vitamin D and vitamin B9, which has been reported previously [14,19]. Vitamin D deficiency has been described in women with PCOS [53], and a growing body of evidence supports vitamin D deficiency in the pathophysiology of PCOS, through mechanisms involving obesity, insulin resistance, hyperandrogenemia, dyslipidemia, ovulatory dysfunction, and inflammation [54,55,56,57,58,59]. Vitamin B9 deficiency has implications in the development of hyperhomocysteinemia and gonadal abnormalities, such as impaired ovarian reserve, and female infertility, beyond the universal agreement about the consequences of neural tube defects [60]. We acknowledge that it is difficult to derive a conclusion about the micronutrient adequacy in both groups based on the recommended dietary allowance values per se. There is a potential to underestimate the dietary intakes of women who meet the estimated average requirement (EAR) cut-offs or overestimate the dietary requirement of women who do not meet the recommended dietary allowance (RDA) or adequate intake (AI) cut-offs due to the specific limitations of these components of dietary recommendation intakes as described previously [61,62].

Unlike others, we did not observe differences in diet quality scores between women with and without PCOS [14]. While there are very few data on the quality of diets consumed by women with PCOS, better diet quality was reported by Moran and colleagues [14]. The discrepancy between studies may stem, in part, from differences in approaches used to define PCOS cohorts. Specifically, Moran et al. identified women with PCOS based on self-reported diagnoses. The presence or absence of PCOS was not clinically verified in neither the PCOS nor the control group, which may have resulted in an under-representation of the PCOS population [14]. It was speculated that better diet quality may have resulted from self-imposed improvements in lifestyle behaviors of women following their knowledge of PCOS diagnosis. Given the cross-sectional nature of studies, it was not possible to address this hypothesis. Ultimately, longitudinal studies are required to evaluate whether women with PCOS adopt certain healthy lifestyle behaviors after receiving a PCOS diagnosis [50].

In addition to dietary recommendations, increasing physical activity is another fundamental strategy to promote weight loss, achieve sustainable weight maintenance, and manage PCOS features and metabolic comorbidities [1]. In the current study, women with PCOS met the recommended national physical activity guidelines [46] as identified by objective and subjective measures. Specifically, women with PCOS engaged in a minimum of 150 min of moderate-intensity aerobic physical activity throughout the week, as evidenced by accelerometry, or reported at least 75 min of vigorous-intensity aerobic physical activity throughout the week, as identified by the Women’s Health Initiative Study Physical Activity Questionnaire [47]. We observed no differences between minutes spent in moderate and vigorous physical activity between women with and without PCOS. These findings were consistent with five other studies that detected no differences in self-reported moderate and vigorous physical activity between women with and without PCOS [12,13,14,15,19]. We are aware of a single study that measured physical activity using accelerometers that noted no significant differences in sedentary levels of obese adolescents with and without PCOS [27], suggesting that physical activity may not differ between women with and without PCOS across the reproductive life span. However, we are unaware of any previous studies that characterized the physical activity of women with PCOS using both objective and subjective measures. In our study, a comparison of subjective and objective physical activity using crossed-matched data in women who completed both the physical activity questionnaire and accelerometry showed a poor level of agreement across specific physical activity intensity levels (data not shown). This level of agreement was not entirely unexpected and may be attributed to recall bias, an overestimation of self-reported physical activity, and technical issues about the use of accelerometers as previously described [63]. Further, it should be noted that the self-reported questionnaire asked for usual physical activity over an extended duration while the accelerometers measured physical activity over a more limited and predefined period [64]. Our observations underscore the need for further research to characterize physical activity patterns in women with PCOS using objective measures wherein little data are available.

This study had several strengths. It was the first to comply with the new diagnostic thresholds to identify PCOS when assessing diet and physical activity behaviors. Our approach was rigorous as we uniformly evaluated polycystic ovarian morphology [25,65] and also used a gold standard methodology for measuring total testosterone concentrations [34]. We acknowledge that the average bilateral FNPO in our controls was slightly higher than the thresholds for polycystic ovaries recommended by the guideline [1]. This observation is consistent with our previous reports of follicle counts in reproductive age women, including those with PCOS, using offline analyses of antral follicle counts, which we have shown to be highly reproducible [25,31,65]. Our detection of a greater number of antral follicles reflects the precision of our methods [25] and its propensity to yield higher antral follicle counts than real-time approaches used in clinical practice. It should be recognized that FNPO in the PCOS group was approximately two-fold higher when compared to controls and none of the controls had increased OV and FNPS, consistent with the morphological differences existing between the groups. Further, our use of the new guideline recommendations to diagnose PCOS increased the external validity of our observations. However, we acknowledge that their use yielded a heterogeneous cohort that limited our ability to ascribe our observations to specific phenotypes of PCOS.

Our study was also the first to use the latest edition of the Dietary Guidelines for Americans’ recommended benchmarks to assess the diet quality of women with PCOS and examine whether they met nationally recommended nutrition needs and physical activity guidelines. These evaluations were particularly informative since these dietary recommendations were created to reduce the risk of chronic disease and acknowledge the role of healthy lifestyle behaviors in achieving this aim [39,41,42,43,46].

Our findings should be interpreted in light of limitations. Subjective assessments of dietary intake may tend toward random or systematic error, recall bias, underreporting, and reactivity [66,67]. Further, the use of FFQ to estimate certain nutrient intakes, such as sodium and fat components, has been criticized [68]. Our study was limited by small sample size and incomplete knowledge of socio-economic status. Many women in the present study were well-educated. Therefore, our observations may be skewed toward responders who had a higher degree of self-awareness and knowledge about their health. Objective assessment of physical activity in the present work may also be too short to capture usual physical activity levels, particularly in sedentary women. Therefore, a longer evaluation of physical activity data using accelerometry is recommended in future work. Our observations about a propensity for obesity in the PCOS group, despite comparable dietary and physical activity behaviors to that of controls, could be attributed to several factors. An altered metabolic rate, in addition to under-reporting of dietary intake due to social desirability or the Hawthorne effect [67], or reverse causation, wherein improvements in dietary intake or quality follow a PCOS diagnosis [69], may have contributed to this observation. However, we cannot make any causal inferences regarding the potential impacts of these factors in the development of obesity or PCOS due to the observational nature of the present study. Future longitudinal research directly assessing the metabolic rate and/or energy expenditure of women across the phenotypic spectrum of PCOS in comparison to their healthy counterparts is needed to fully elaborate this question as current evidence in this area remains controversial [70,71,72,73].

## 5. Conclusions

Our findings support and extend previous observations that confirm the lack of substantial differences in dietary and physical activity behaviors between women with and without PCOS. Our observations reiterate the feasibility of the healthy lifestyle recommendations proposed in the recent International Evidence-based Guideline for the Assessment and Management of PCOS as we did not identify unique targets of diet or physical activity intervention. They also highlight the important role of nutrition professionals to provide evidence-based healthcare to women with PCOS to assist them in meeting targets for healthy lifestyle practices and making informed decisions about improving their short- and long-term reproductive and metabolic health [5,52,74].

## Figures and Tables

**Table 1:** Demographic, anthropometric, clinical, biochemical, and ultrasonographic characteristics of women with polycystic ovary syndrome and controls.

Measure (unit)	All Women ^a^	PCOS ^a^	Control ^a^	Reference	*p*-Value
Age (year)	27.7 (26.6–28.8)	26.8 (25.4–28.1)	29.5 (27.5–31.4)	N/A	0.02
Ethnicity, Hispanic (n (%))	18 (14.5)	9 (11.3)	9 (20.5)	N/A	0.10
Race (n (%))				N/A	0.15
Black	15 (12)	8 (10)	7 (16)		
Asian	11 (9)	7 (9)	4 (9)		
White	77 (62)	55 (69)	22 (50)		
Other	21 (17)	10 (12)	11 (25)		
Metformin use (n (yes %))	1 (1)	1 (1)	0 (0)	N/A	1.00
OCP use (n (yes %))	7 (6)	4 (5)	3 (7)	N/A	0.70
BMI (kg/m^2^)	30.2 (28.8–31.6)	31.5 (29.5–33.4)	28.0 (26.1–29.8)	18.5–24.9	0.01
Normal BMI (n (yes %))	42 (33.9)	24 (30.0)	18 (40.0)	18.5–24.9	
Overweight (n (yes %))	20 (16.1)	11 (13.8)	9 (20.4)	25.0–29.9	0.19
Obese (n (yes %))	62 (50.0)	45 (56.2)	17 (38.6)	≥30.0	
WHR	0.84 (0.82–0.85)	0.83 (0.81–0.85)	0.84 (0.83–0.86)	≤0.85	0.49
Menstrual cycle length (d)	64 (49–78)	86 (63–108)	29.5 (26.7–32.3)	<36	0.001
Modified hirsutism score	6 (5–7)	8 (6–9)	4 (3–5)	<6	<0.0001
TT (nmol/L)	1.6 (1.4–1.7)	1.8 (1.6–1.9)	1.2 (1.1–1.4)	<2.1	<0.0001
FAI	4 (4–5)	6 (6–6)	2 (2–3)	<6	0.001
Free T (nmol/L)	0.02 (0.02–0.03)	0.03 (0.03–0.03)	0.02 (0.01–0.02)	<0.03	<0.0001
Bioavailable T (nmol/L)	0.6 (0.5–0.6)	0.7 (0.6–0.8)	0.4 (0.3–0.4)	<0.7	<0.0001
OV (mL)	9.9 (8.9–10.9)	11.0 (9.7–12.2)	7.8 (6.3–9.4)	<10	<0.004
FNPS (n)	9 (8–10)	10 (9–11)	7 (6–8)	<10	<0.005
FNPO 2–9 mm (*n*)	36 (32–40)	42 (36–47)	24 (20–28)	<20	<0.0001

Abbreviations: PCOS, polycystic ovary syndrome; OCP, oral contraceptive pills; BMI, body mass index; WHR, waist to hip ratio; TT, total testosterone; FAI, free androgen index; OV, ovarian volume; FNPS, follicle number per section; FNPO, follicle number per ovary. ^a^ Data are expressed as mean (95% confidence interval) or numbers (percentages). Demographic, anthropometric, clinical, biochemical, and ultrasonographic characteristics were measured for *n* = 124 women (*n* = 80 in the PCOS and *n* = 44 in the control groups).

**Table 2:** Dietary intake and diet quality of women with polycystic ovary syndrome and controls.

Measure (unit)	All Women ^a^	PCOS ^a^	Control ^a^	Reference	*p*-Value
**Dietary factors ^b^**					
Energy (kcal/d)	2204 (2036–2373)	2218 (2017–2419)	2180 (1866–2494)	2403 ^c^	0.64
Total carbohydrate (g/d)	267 (245–290)	264 (240–288)	273 (225–321)	100 ^d^	0.70
Added sugars (g/d)	70 (59–82)	68 (56–80)	75 (52–99)	N/A	0.64
Total protein (g/d)	85 (79–92)	86 (78–95)	83 (72–94)	46 ^e^	0.60
Total fat (g/d)	87 (80–94)	89 (80–99)	83 (72–94)	N/A	0.42
Total SFA (g/d)	28 (26–31)	29 (25–32)	28 (23–33)	N/A	0.77
Total MUFA (g/d)	34 (33–37)	35 (31–39)	32 (28–36)	N/A	0.40
Total PUFA (g/d)	18 (16–19)	18 (16–20)	17 (15–19)	N/A	0.37
Cholesterol (mg/d)	291 (262–321)	303 (264–342)	271 (229–314)	N/A	0.34
Trans fats (g/d)	3 (3–3)	3 (3–4)	3 (2–3)	N/A	0.94
Total fiber (g/d)	23 (22–26)	24 (22–26)	25 (22–28)	28 ^f^	0.49
Vitamin A (IU/d)	14511 (12634–16388)	13933 (117167–16150)	15561 (12029–19094)	500 ^d^	0.60
Vitamin B_1_ (mg/d)	1.8 (1.7–1.9)	1.8 (1.7–2.0)	1.8 (1.5–2.0)	0.9 ^d^	0.46
Vitamin B_2_ (mg/d)	2.4 (2.2–2.6)	2.4 (2.2–2.6)	2.3 (2.0–2.7)	0.9 ^d^	0.27
Vitamin B_3_ (mg/d)	24 (22–25)	24 (22–26)	22 (19–25)	11 ^d^	0.15
Vitamin B_5_ (mg/d)	7 (6–7)	7 (6–7)	7 (6–8)	5 ^h^	0.37
Vitamin B_6_ (mg/d)	2.2 (2.0–2.3)	2.2 (2.1–2.4)	2.1 (1.8–2.3)	1.1 ^d^	0.28
Vitamin B_9_ (μg/d)	285 (265–305)	275 (252–298)	303 (265–340)	320 ^d^	0.38
Vitamin B_12_ (μg/d)	5.7 (5.1–6.3)	5.9 (5.2–6.6)	5.3 (4.3–6.3)	2.0 ^d^	0.17
Vitamin C (mg/d)	141 (125–157)	136 (118–154)	151 (120–181)	60 ^d^	0.55
Vitamin D (μg/d)	6 (5–7)	6 (5–7)	6 (4–7)	10 ^d^	0.48
Vitamin E (mg/d)	21 (19–22)	21 (19–23)	20 (17–23)	12 ^d^	0.60
Vitamin K (μ/d)	219 (190–251)	197 (166–228)	259 (188–331)	90 ^h^	0.11
Calcium (mg/d)	1141 (1033–1250)	1117 (997–1236)	1187 (967–1407)	800 ^d^	0.89
Copper (μg/d)	1536 (1441–1630)	1516 (1407–1624)	1572 (1387–1758)	700 ^d^	0.57
Iron (mg/d)	16 (15–18)	17 (15–18)	16 (14–18)	8.1 ^d^	0.54
Magnesium (mg/d)	363 (340–386)	357 (331–383)	375 (330–420)	255–265 ^d^	0.46
Manganese (mg/d)	4.5 (4.1–5.0)	4.3 (3.9–4.6)	5.0 (4.0–6.0)	1.8 ^g^	0.27
Phosphorus (mg/d)	1439 (1130–1548)	1445 (1311–1580)	1427 (1235–1618)	580 ^e^	0.87
Potassium (g/d)	3.2 (3.0–3.5)	3.2 (2.9–3.5)	3.3 (2.8–3.8)	2.6 ^g^	0.68
Selenium (μg/d)	123 (114–133)	126 (114–138)	118 (103–134)	45 ^d^	0.45
Sodium (g/d)	4.0 (3.7–4.3)	4.0 (3.6–4.4)	4.1 (3.5–4.7)	2.3 ^h^	0.75
Zinc (mg/d)	13 (12–14)	13 (11–14)	13 (11–14)	6.8 ^d^	0.44
Caffeine (mg/d)	165 (138–192)	151 (123–180)	191 (135–247)	N/A	0.46
Alcohol (g/d)	9 (7–11)	9 (5–12)	9 (5–13)	N/A	0.58
**HEI-2015 components ^b^**				Maximum points ^i^	
Total fruits	3.6 (3.3–3.9)	3.5 (3.1–3.9)	3.7 (3.2–4.2)	5	0.59
Whole fruits	3.9 (3.6–4.2)	3.9 (3.5–4.2)	3.9 (3.4–4.4)	5	0.98
Total vegetables	4.2 (4.0–4.4)	4.2 (3.9–4.4)	4.3 (4–4.6)	5	0.67
Greens and beans	3.8 (3.5–4.0)	3.7 (3.3–4.1)	3.9 (3.4–4.4)	5	0.51
Whole grains	4.9 (4.3–5.3)	4.0 (5.3–4.6)	5.2 (4.3–6.1)	10	0.31
Dairy	6.2 (5.7–6.6)	6.1 (5.5–6.7)	6.4 (5.6–7.2)	10	0.55
Total protein foods	4.6 (4.4–4.7)	4.5 (4.3–4.7)	4.6 (4.4–4.9)	5	0.40
Seafood and plant proteins	4.3 (4.0–4.5)	4.1 (3.8–4.4)	4.5 (4.2–4.8)	5	0.12
Fatty acids	5.3 (4.8–5.9)	5.5 (4.8–6.3)	4.9 (4.1–5.8)	10	0.39
Refined grains	8.3 (7.9–8.7)	8.3 (7.6–8.8)	8.3 (7.6–9.1)	10	0.49
Sodium	2.9 (2.4–3.3)	3.0 (2.4–3.5)	2.7 (2.0–3.5)	10	0.49
Added sugars	8.3 (7.9–8.7)	8.4 (7.9–8.8)	8.2 (7.5–8.7)	10	0.74
Saturated fats	5.9 (5.4–6.4)	5.9 (5.2–6.6)	5.8 (5.0–6.6)	10	0.86
Total HEI-2015 score	65.9 (63.7–68.1)	65.6 (62.6–68.6)	66.5 (63.5–69.6)	100 ^i^	0.70

Abbreviations: PCOS, polycystic ovary syndrome; SFA, saturated fatty acid; MUFA, monounsaturated fatty acids; PUFA, polyunsaturated fatty acids; HEI, Healthy Eating Index. ^a^ Data are expressed as mean (95% confidence interval). ^b^ Dietary factors and diet quality were measured for *n* = 124 women (*n* = 80 in the PCOS and *n* = 44 in the control groups). ^c^ Dietary Reference Intakes (DRIs; (taken from the DRI reports, see www.nap.edu) for healthy active Americans (19 years of age) at the reference heights and weight. Subtract 7 kcal/d for women for each year of age above 19 years. ^d^ Estimated Average Requirements (taken from the DRI reports, see www.nap.edu). A higher dietary intake may be advisable to meet the required dietary intake of individuals. ^e^ Recommended Dietary Allowances (taken from the DRI reports, see www.nap.edu). Recommended cut-off values may be used as goals for individual dietary intake. ^f^ 14 g/1000 kcal, based on the 2015–2020 Dietary Guidelines for Americans [39]. Recommended cut-off values may be used as goals for individual dietary intake. ^g^ Adequate Intakes (taken from the DRI reports, see www.nap.edu). Recommended cut-off values may be used as goals for individual dietary intake. ^h^ Chronic Disease Risk Reduction Intake (taken from the DRI reports, see www.nap.edu). A dietary intake lower than the cut-off value is recommended for the healthy population. ^i^ HEI-2015 scores were measured as described [41,42,43]. A higher score represents a better diet quality. Dietary intakes were not different between groups after adjusting for age and body mass index differences.

## Supplementary Materials

The following are available online at https://www.mdpi.com/2072-6643/11/11/2711/s1, Table S1: Physical activity levels of women with polycystic ovary syndrome and controls who completed both the accelerometry and self-reported physical activity questionnaire.
