# Supplementary material for: Dietary and Physical Activity Behaviors in Women with Polycystic Ovary Syndrome per the New International Evidence-Based Guideline
Source: Nutrients. 2019 Nov 8;11(11):2711. doi: 10.3390/nu11112711 (PMC6893689; doi:10.3390/nu11112711)
Supplement: Supplementary file 1 [file nutrients-11-02711-s001.pdf]

Table S1. Physical activity levels of women with polycystic ovary syndrome and controls who completed both the accelerometry and self-reported physical activity questionnaire.

Measure (unit)	All women [a]	PCOS [a]	Control [a]	Reference	*p*-Value
**Physical activity levels determined by accelerometer** [b]					
Sedentary and light activity (h/wk)	65 (65 – 66)	65 (65 – 66)	64 (64 – 66)	N/A	0.45
Moderate physical activity (mins/wk)	260 (233 – 286)	244 (211 – 277)	282 (236 – 327)	150 [c]	0.17
Vigorous physical activity (mins/wk)	35 (25 – 45)	40 (26 – 55)	27 (15 – 38)	75 [c]	0.19
Sedentary and light activity (MET-h/wk)	79 (77 – 81)	78 (76 – 81)	79 (76 – 82)	N/A	0.87
Moderate physical activity (MET-h/wk)	20 (18 – 23)	20 (16 – 24)	21 (17 – 26)	N/A	0.59
Vigorous physical activity (MET-h/wk)	1 (1 – 2)	1 (1 – 2)	1 (0 – 2)	N/A	0.31
**Self-reported physical activity levels** [b]					
Sitting and lying down (h/wk)	49 (49 – 56)	49 (49 – 56)	49 (49 – 56)	N/A	0.62
Walking physical activity (min/wk)	127 (108 – 146)	133 (104 – 161)	119 (94 – 144)	N/A	0.44
Mild physical activity (min/wk)	27 (17 – 37)	24 (11 – 36)	31 (13 – 48)	N/A	0.66
Moderate physical activity (mins/wk)	63 (48 – 79)	64 (43 – 86)	62 (40 – 84)	150 [c]	0.89
Vigorous physical activity (mins/wk)	87 (63 – 110)	93 (56 – 126)	79 (46 – 111)	75 [c]	0.92
Walking physical activity (MET-h/wk)	3 (2 – 3)	3 (2 – 3)	2 (2 – 3)	N/A	0.44
Mild physical activity (MET-h/wk)	1 (1– 2)	1 (1 – 1)	2 (0 – 2)	N/A	0.66
Moderate physical activity (MET-h/wk)	5 (4 – 6)	5 (3 – 6)	5 (3 – 6)	N/A	0.89
Vigorous physical activity (MET-h/wk)	10 (7 – 13)	11 (7 – 15)	9 (5 – 13)	N/A	0.92

[a] Data are expressed as mean (95% confidence interval).
[b] Physical activity levels were measured for n=75 women (n=44 in the PCOS and n=31 in the Control groups).
[c] Minimum levels of moderate- or vigorous-intensity recommended aerobic activity levels per week for substantial health benefits, as per recommended by the 2018 International Guidelines for the Assessment and Management of polycystic ovary syndrome [1] and the 2018 Physical Activity Guidelines for Americans [2].
Student t-test or Mann-Whitney U test was used to compare physical activity levels between groups. Physical activity levels were not different between groups after adjusting for age and body mass index differences using the analysis of covariance.

References

1. Teede, H.J.; Misso, M.L.; Costello, M.F.; Dokras, A.; Laven, J.; Moran, L.; Piltonen, T.; Norman, R.J. Recommendations from the international evidence-based guideline for the assessment and management of polycystic ovary syndrome. *Hum Reprod* **2018**, *33*, 1602-1618, doi:10.1093/humrep/dey256.
2. Piercy, K.L.; Troiano, R.P.; Ballard, R.M.; Carlson, S.A.; Fulton, J.E.; Galuska, D.A.; George, S.M.; Olson, R.D. The Physical Activity Guidelines for Americans. *JAMA* **2018**, *320*, 2020-2028, doi:10.1001/jama.2018.14854.
